# Re: Rates of out-of-home care among children in Canada: an analysis of national administrative child welfare data

**DOI:** 10.24095/hpcdp.44.5.05

**Published:** 2024-05

**Authors:** Chandrakant P. Shah

**Affiliations:** 1 Professor Emeritus, Dalla Lana School of Public Health, University of Toronto, Toronto, Ontario, Canada; 2 Hon. Consultant, Anishnawbe Health Toronto, Toronto, Ontario, Canada

Dear Editor,

I recently read in your journal the article by Pollock et al. titled “Rates of Out-of-Home Care Among Children in Canada: An Analysis of National Administrative Child Welfare Data.”^1^ I commend the authors for their work. For the past 40years, the public health and health care professions have increasingly paid attention to the social determinants of health and the upstream approaches to prevention; however, they have neglected children in out-of-home care for too long, as evidenced by the paucity of literature on this vulnerable population. I believe that the majority of the public, including health professionals, assumed that the children who were removed from their homes and placed under the care of government-appointed agencies (mostly Children’s Aid Societies or some form of group home) were generally well looked after in terms of their mental, physical and cognitive health. However, this view is not always supported by evidence,^2^ and my own experiences also tell a different story.

In the early 1970s, when I was the inaugural full-time medical director for the Children’s Aid Society of Vancouver, I was shocked by what I learned. I conducted several studies and wrote papers outlining the dire situation of these vulnerable children and proposed solutions. For example, one-third of the children coming into care had physical, emotional or cognitive disabilities.^3^-^5^ There was a shortage of habilitation services in their communities, which forced children with disabilities to be admitted to foster care. Still, no health histories were available when the children were admitted into care, and there were no transmissions of health histories back to their primary care physicians when they were discharged. There was no uniform system of payment, a shortage of foster homes and a lack of training for foster families caring for children with disabilities, leading to frequent breakdowns of placement and resulting in some children having to move from one foster home to another 10 to 12 times in as little as 12 months.^6^ In those days, about 30–40% of children in care were Indigenous and living far from their communities in non-Indigenous foster homes. Thanks to the campaign spearheaded by Dr. Cindy Blackstock, the Executive Director of the First Nations Child and Family Caring Society of Canada, Indigenous children in need of foster care are now being looked after in their home communities. However, due to the lack of facilities in the Indigenous communities in north, many youths with mental health issues are still being placed in private group homes, hundreds of kilometers south away from their communities and are considered by some group homeowners as ‘money-makers,’ as recently reported by Global News.^7^

Have things changed over the last 50years? At least the number of children receiving out-of-home care has gone down from approximately 100000 to 61000.1 However, are they receiving appropriate care for their needs? I cannot answer that, but I doubt it. But we now have a unique opportunity to address this issue by linking administrative data from child welfare agencies to our health insurance database to evaluate our programs. I challenge the authors and others in the field to expand their research and enlighten us all on the plight of these most vulnerable children! As an 87-year-old, I know that it is time for us to act.
